# Enterotoxins: Microbial Proteins and Host Cell Dysregulation

**DOI:** 10.3390/toxins8010017

**Published:** 2016-01-08

**Authors:** Teresa Krakauer

**Affiliations:** Department of Immunology, Molecular Translational Sciences Division, United States Army Medical Research Institute of Infectious Diseases, Fort Detrick, Frederick, MD 21702 5011, USA; Teresa.krakauer.civ@mail.mil; Tel.: +1-301-619-4733

**Keywords:** staphylococcal superantigens, clostridial toxins, receptor binding, toxicity, inflammation, therapeutics

The special issue “Enterotoxins: Microbial Proteins and Host Cell Dysregulation” is comprised of research articles and reviews covering a diverse group of toxins that affect the gut and dysregulate host immune response in mechanistically different ways. Excellent in-depth reviews of staphylococcal superantigens and *Clostridium perfringens* toxins are the cornerstones of this issue. The present editorial highlights these papers grouped by toxin class and within each toxin class papers are discussed in order of publication date, with reviews appearing first, followed by original articles.

The review entitled “Update on staphylococcal superantigen-induced signaling pathways and therapeutic interventions” gives a comprehensive review of signaling pathways induced by staphylococcal superantigens and evaluations of therapeutics targeting these pathways [1]. Of great interest is the presentation of pathways engaged by these bacterial superantigens, an extensive list of effective and ineffective therapies, as well as the benefits associated with specific targeting of activated molecules and pathways. A detailed pathway diagram of signaling molecules and points of intervention by specific inhibitors is available to readers.

Another information-rich review, “Soluble T cell receptor Vβ domains engineered for high-affinity binding to staphylococcal or streptococcal superantigens”, presents a brief review of the structure and sequence homology of multiple superantigens produced by *Staphylococcus aureus* and group A *Streptococcus* [2]. The different modes of binding of these superantigens to both major histocompatibility complex (MHC) class II and variable regions of T cell receptor beta chain (TCR Vβ) are presented, including the targeting of specific epitopes on superantigens with high affinity TCR Vβ mutants. A flow chart and schematic of yeast display methodology illustrate techniques used in the engineering of these soluble high affinity TCR Vβ domains against superantigens. The interaction of each specific high affinity Vβ domain against the superantigens SEA, SEB, SEC3, TSST-1, SpeA, and SpeC is displayed by their co-crystal structures. Of great interest to the scientific community is the utility of these high affinity TCR Vβ domains in blocking superantigen-induced lethality and preventing necrotizing pneumonia induced by SEC secreting methicillin-resistant *S. aureus* in various rabbit models of disease.

The review article “Staphylococcal enterotoxins in the etiopathogenesis of mucosal autoimmunity within the gastrointestinal tract” addresses the immunopathogenic effects of staphylococcal enterotoxins in the gastrointestinal tract [3]. Different immune cell types, Th1, Th2, Th17 and regulatory T cells, participating in gut immune defense and tolerance are described. The early activation of specific Vβ T cells in blood and lymphoid organs results in induction of proinflammatory mediators (IL-1β, IL-2, IL-6, IL-8, MCP-1, IFNγ and TNFα) eliciting inflammatory cell infiltration to the gastrointestinal tract and the proliferation of T cells in lymphoid organs. SEB also directly reduces mucosal tight-junction proteins and, together with SEB-induced inflammatory cytokines, destroys epithelial barriers in the intestine, thus initiating pathological effects. The histochemical presentation of the structural destruction of mucosal tissues and cells illustrates the induction of apoptosis by SEB in mouse Peyer’s patches after oral gavage of the toxin. Valuable information is provided in this review regarding the contribution of regulatory T cells, IL-10 and TGFβ in immune tolerance induction by staphylococcal enterotoxins.

Three articles describe therapeutics that are effective in attenuating the biological effects of staphylococcal superantigens in this special issue. Three different approaches using “novel inhibitor” [4], an FDA-approved drug [5], or intravenous immunoglobulin (IVIG) therapy [6] are presented, addressing the urgent need for developing therapeutics against staphylococcal superantigens. The article “Treatment with the hyaluronic acid synthesis inhibitor 4-methylumbelliferone suppresses SEB-induced lung inflammation” presents the use of 4-methylumbelliferone (4-MU), an inhibitor of hyaluronic acid (HA) synthesis, to diminish SEB-induced lung inflammation *in vivo* [4]. *In vitro* studies using mouse splenocytes showed 4-MU inhibits SEB-induced T cell apoptosis and downregulates cytokine expression. Data presented suggest that specificity lies in the ability of 4-MU to suppress SEB-induced hyaluronic acid synthase and accumulation of soluble HA. In addition, the authors show that 4-MU protects mice from SEB-induced acute lung injury as measured by decreases in vascular permeability in mouse lungs.

The article “Toxic shock syndrome toxin-1-mediated toxicity inhibited by neutralizing antibodies late in the course of continual *in vivo* and *in vitro* exposure” describes the use of neutralizing antibodies to suppress staphylococcal superantigen TSST-1-induced lethal shock in the rabbit [6]. An important take-home message from this article is that neutralizing antibodies are effective even when administered at late stages after toxin encounter in this animal model. Hyperimmune antiserum inhibits both IL-2 and TNFα *in vitro* using human peripheral blood mononuclear cells (PBMC). The article illustrates that hyperimmune antiserum obtained from repeated immunization contains high affinity antibodies capable of neutralizing continued T cell activation in a new TSST-1 infusion model. It would be interesting to test the specificity of hyperimmune antiserum and its applicability in human toxic shock.

A short report, “Sulfasalazine attenuates staphylococcal enterotoxin B-induced immune responses” evaluates an FDA-approved arthritis drug, sulfasalazine, in suppressing SEB-induced T cell proliferation and cytokine production in SEB-stimulated human PBMC [5]. Sulfasalazine acts as an NFκB inhibitor in attenuating SEB-induced effects *in vitro*.

The article “Assessment of the functional regions of the superantigen staphylococcal enterotoxin B” provides a different approach in determining the structural regions of SEB involved in its functional activity [7]. The functional activity of *N*- and *C*-terminal truncations of SEB fused to bacterial-binding protein was determined by assessing mitogenic activity, MHC class II binding, and TCR Vβ selectivity. Although a specific epithelial membrane protein has not been identified, the suggestion by the authors that a non-productive TCR response is produced by SEB binding to a putative epithelial cell membrane protein is intriguing.

The paper entitled “Superantigens modulate bacterial density during *Staphylococcus aureus* nasal colonization” addresses an important topic of the contribution of staphylococcal superantigens in nasal colonization by *S. aureus* [8]. A new nasal colonization model using superantigen-sensitive HLA-DR4 transgenic mice is presented to evaluate bacterial strains *S. aureus* Newman, *S. aureus* Newman *Δsea*, *S. aureus* COL, and *S. aureus* COL *Δseb* in colonization. Data indicate that only *S. aureus* COL *Δseb* maintains a higher bacterial density throughout the duration of the study and neither SEA nor SEB are involved in bacterial dissemination from the nasal cavity. The authors conclude that the inflammatory properties of SEB likely increase colonization and superantigens modulate bacterial density during colonization.

The review entitled “*Clostridium perfringens* epsilon toxin: a malevolent molecule for animals and man?” starts with a brief review of alpha, beta and iota toxins from *C. perfringens* [9]. This is followed by a comprehensive review of epsilon toxin (Etx) produced by *C. perfringens* (types B and D), its mechanism of action and enteric effects, as well as therapeutic countermeasures. Disease associated with Etx occurs in animals as enterotoxemia, as Etx induces pore formation in eukaryotic cell membranes. The authors discuss the importance of the disruption of membrane integrity of various organs by Etx, which promotes perivascular leakage of proteins, ion efflux, cell damage and death. Readers are encouraged to read the various animal models and therapeutic development presented in this review.

A different pore-forming toxin, beta-toxin from *C. perfringens*, is reviewed in “Recent insights into *Clostridium perfringens* beta-toxin” [10]. *C. perfringens* type C strains induce food-borne necrotizing enterocolitis in humans and lethal infections in animals (neonates of pigs, cattle, goats, sheep) where bacteria propagate in the small bowel and produce toxins. Different animal models are presented to study the biological effects of beta-toxin. The focus of this paper is on beta-toxin binding to cells and its biological effects in different animal models. Beta-toxin binding to vascular endothelial cells causes vascular necrosis in the piglet jejunal loop model. As beta-toxin attacks both central and peripheral nerves, the release of substance P and TNFα results in plasma extravasation. Beta-toxin binding to specific receptors in lipid rafts of immune cells promotes potassium efflux and activates various MAP-kinases.

The article entitled “Binding studies on isolated porcine small intestinal mucosa and *in vitro* toxicity studies reveal lack of effect of *C. perfringens* beta-toxin on the porcine intestinal epithelium” investigates the binding of *C. perfringens* beta-toxin to epithelial and endothelial cells using porcine neonateal jejunal explants and cryosections [11]. Contrary to other studies, the data presented suggest that beta-toxin does not induce cytotoxicity in cultured porcine intestinal epithelial cells and fails to bind these epithelial cells.

A review of binary enterotoxins presented in “*Clostridium and Bacillus* binary enterotoxins: bad for the bowels, and eukaryotic being” adds further knowledge of multiple bacterial enterotoxins [12]. The binary toxins from *C. botulinum*, *C. difficile*, *C. perfringens*, *C. spiroforme* and *B. cereus* are gut-acting proteins consisting of two components, an ADP-ribosyl transferase (component A) and cell-binding component B. Details of protein structure, mechanism of action and pathogenic effects of the different types of binary enterotoxins, from the well-known C2 toxin to the relatively under-studied *C. spiroforme* toxin, are presented. Of interest is the presentation of a proposed mechanism on the unfolding of the A components of *Clostridium* and *Bacillus* binary toxin which is then “thread through toxin-generated channels in membranes into the cytosol”. Readers are encouraged to read this idea- and information-packed review of how different gut-acting bacterial binary toxins intoxicate cells, starting with damage of the cytoskeleton.

The paper entitled “The combined repetitive oligopeptides of *Clostridium difficile* toxin A counteract premature cleavage of the glucosyl-transferase domain by stabilizing protein conformation” presents original data of the mechanistic interaction of toxin domains of *C. difficile* that promotes toxin A stability [13]. This article presents a new concept of domain binding which then induces protein stability in *C. difficile* toxin A. Various functional domains in the autoproteolytic processing and inactivation of toxin A were measured. The authors conclude that the C-terminally-located combined repetitive oligopeptides are responsible for protein conformation and stability as well as the receptor binding of toxin A.

The article “*Clostridium perfringens* enterotoxin (CPE) and CPE-binding domain (c-CPE) for the detection and treatment of gynecologic cancers” reviews the use of “receptors” claudin-3 and claudin-4, which bind CPE, to detect and treat certain gynecologic tumors [14]. Of interest is the translational application of using labeled c-CPE peptide for detection of rare metastatic tumors in the clinic.

The paper “Cholera toxin B: one subunit with many pharmaceutical applications” reviews the biological applications of cholera toxin B (CTB) [15]. The authors summarize evidence of immunological activities of CTB that can be utilized by the toxin. Various animal models using CTB as an adjuvant are presented. However, translational application to humans is questionable as CTB cannot be used in humans as a nasal adjuvant due to the risk of the development of facial paralysis.

The paper entitled “Do the A subunits contribute to the differences in the toxicity of Shiga toxin 1 and Shiga toxin 2?” presents a comparative review of a different class of enteric toxins from the enterovirulent *E. coli* family [16]. Structural and functional differences of the two toxins Shiga toxin 1 (Stx1) and Shiga toxin 2 (Stx2) are presented. The two toxins have differential toxicity on endothelial cell lines. The catalytic activities and interactions with ribosomes of Stx1 and Stx2 are compared to ricin in this article. Although Stx1 is more inflammatory and, perhaps as a consequence, induces more severe hemorrhagic enterocolitis, controversies exist in the literature regarding the toxic potential of these two toxins in different cells and animal models.
